# UK corneal surgeons’ attitudes towards splitting donor corneas between multiple recipients

**DOI:** 10.1038/s41433-024-03556-6

**Published:** 2024-12-20

**Authors:** Jamie A. Yule, Virginija Vilkelyte, Shakeel Ahmad, Poonam Sharma, James Myerscough, Stephen Kaye, Harry W. Roberts

**Affiliations:** 1 General Surgery, Ealing Hospital, West London, UK; 2 University of Exeter Medical School, Exeter, UK; 3 West of England Eye Unit, Royal Devon University Healthcare NHS Foundation Trust, Exeter, UK; 4 Department of Ophthalmology, Southend University Hospital, Southend-on-Sea, UK; 5 Faculty of Health, University of Plymouth, Plymouth, UK; 6 Department of Eye and Vision Science, University of Liverpool, Liverpool, UK

**Keywords:** Outcomes research, Health services

Keratoplasty is one of the most widely performed and highly effective transplant procedures worldwide [1]. Unfortunately, this procedure is met by a longstanding scarcity of donor corneas with an estimated deficit of 1500 per year even prior to the COVID-19 pandemic [2]. In October 2017, NHS Blood and Transplant (NHSBT) noted that the supplies in its eye banks were deficient by 21% compared to the required level [3]. Two years later, the situation had not shown any improvement [4]. The Covid-19 pandemic has only worsened this deficit with many patients, at the time of writing, waiting more than 18 months for elective transplants on the NHS [5].

Advances in lamellar surgery offer a compelling solution to the escalating supply issues in the UK [6]. Penetrating keratoplasty (PK) is no longer the preferred technique for most corneal transplants and it is estimated that 80% of patients requiring transplants can be managed with an anterior lamellar keratoplasty (ALK) or an endothelial keratoplasty (EK) [7]. These procedures transplant either the anterior or posterior corneal layers; the remaining lamella could be used to treat another patient [6].

Dividing a single organ among multiple recipients includes well-established practices such as segmental liver transplants [8, 9] and the extraction of donor sclera from the corneal button, which is then divided into patch grafts for glaucoma procedures [10, 11]. Nevertheless, routine division of the cornea itself remains uncommon in the UK, attributed to the convention of allocating donor corneas to individual recipients, driven in part by the ease of traceability [5]. However, this practice results in underutilisation of the remaining layers of corneal tissue, which is disconcerting given the scarcity of available donor tissue. In contrast, this technique is more commonly practiced in other countries such as Germany [12], India [13], and Canada [14].

In April 2023 an anonymous online survey was distributed to all 264 members of the Bowman Club, which comprises consultant corneal surgeons practising in the UK and invited corneal fellows. This survey explored the attitudes of UK corneal surgeons towards splitting donor material and the perceived barriers, given what is already known from the literature. The survey was designed in Microsoft Forms and included five questions on corneal graft supply, splitting tissue and the obstacles to doing so. No demographic data were collected. A reminder e-mail was circulated after two weeks.

The survey closed after three weeks with 54 responses, a response rate of 20%. A sample size of 157 would be needed to have a confidence level of 95% that our results are within 5% of the true value.

The survey confirms a significant shortage of donor corneas, with 90.7% of consultants agreeing on the shortage, 83.3% strongly agreeing. Overall, 92.5% of clinicians were in favour of splitting corneal tissue. Of this, the majority (75.5%) supported the practice in both elective and emergency cases while 7.5% restricted it to elective procedures, and 9.4% recommended it only for emergencies, i.e. where residual tissue from an elective case is used for therapeutic or tectonic purposes in emergency surgery.

The survey also assessed surgeons’ experience with corneal tissue splitting: 74.1% of respondents had split no corneas in the past 12 months and only two surgeons had split tissue more than 5 times.

Ten potential obstacles to splitting corneas, derived by the authors, were offered to respondents who were asked to select one or more (Fig. 1). The three most common barriers are logistical difficulties (69.2%), lack of regulatory support (67.3%) and concerns about infection (35.8%) (percentages of the total number of options chosen). Logistical difficulties include inadequate storage facilities, lack of theatre time and demand for posterior grafts exceeding anterior grafts [15].

**Fig. 1:**
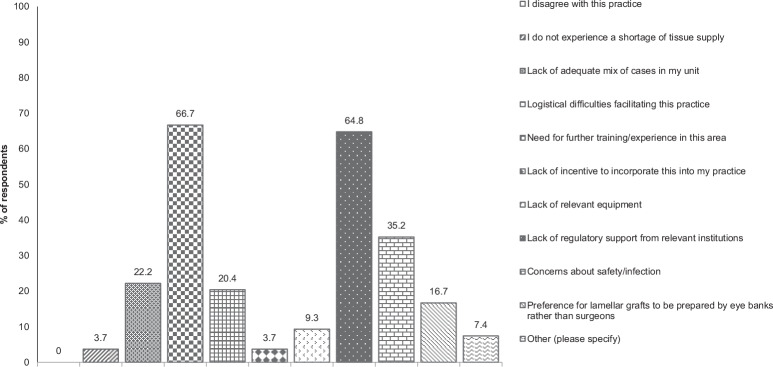
Bar graph of respondents’ answers to: What are the barriers to splitting donor corneas for multiple recipients in your practice? (Please tick all that apply).

Finally, the preferred methods of splitting corneal material were explored (Fig. 2). Respondents were asked to select one or more from a list of five. The most popular options were splitting a corneoscleral button into grafts for DMEK and DALK procedures (86.8%) and using residual tissue from an elective procedure for therapeutic or tectonic purposes (67.0%).

**Fig. 2:**
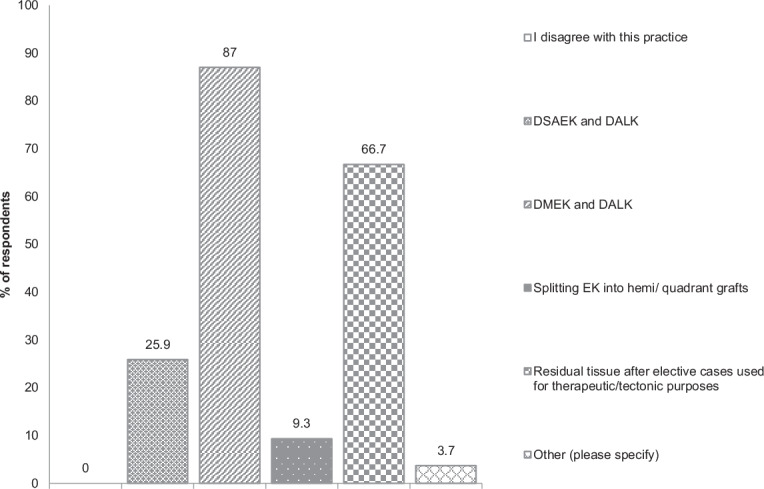
Bar graph of respondents’ answers to: In which ways would you prefer to split grafts? (Please tick all that apply).

Interestingly, our survey reveals overwhelming support for splitting corneas, despite a notable lack of current experience in the UK with this practice. This discrepancy suggests that while surgeons favour the idea of splitting corneas, various factors prevent them from implementing it. Survey responses highlighted the critical role of eye banks, particularly in providing pre-cut grafts that ensure both reliability and convenience. It is essential from a regulatory perspective that eye banks manage the potential risks associated with splitting tissue, such as disease transmission. Furthermore, having eye banks handle tissue splitting enhances the traceability of human tissue, a process supported by the latest version of the NHSBT corneal transplant audit form (FRM4314/2.1), which includes the capability to record when corneas are split.

Previous studies demonstrate that corneal splitting is a feasible and safe option, with one cornea benefitting up to five patients by separating it into four quarter DMEKs and one DALK [16, 17]. Utilising donor tissue in this way would optimise the utilisation of existing resources whilst bridging the demand-supply gap between available donor tissues and patients awaiting keratoplasties.

By investing in specialist storage equipment and meeting HTA licensing regulations, ophthalmology departments could split tissues themselves and preserve the lamellae for seven days [18, 19]. Additionally, by strategically listing an ALK and EK on the same theatre session one donor cornea could be used for multiple patients [6, 18]. The logistics and administrative burden of this practice are significant and therefore this approach is adopted by only a few UK centres.

Despite eye banks being best equipped to split corneas, the reality is that UK corneal surgeons cannot consistently rely on UK eye banks to provide lamellar grafts punctually in contrast to eye banks in other countries which regularly provide pre-cut, pre-stripped or pre-loaded tissues. Utilising each cornea for multiple recipients would alleviate the burden on UK eye banks and hopefully improve their reliability, as well as combating the current deficit in donor corneas.

## Data Availability

Data available upon written request to the corresponding author.
