# Correction: UK corneal surgeons’ attitudes towards splitting donor corneas between multiple recipients

**DOI:** 10.1038/s41433-025-03695-4

**Published:** 2025-03-04

**Authors:** Jamie A. Yule, Virginija Vilkelyte, Shakeel Ahmad, Poonam Sharma, James Myerscough, Stephen Kaye, Harry W. Roberts

**Affiliations:** 1 General Surgery, Ealing Hospital, West London, UK; 2 University of Exeter Medical School, Exeter, UK; 3 West of England Eye Unit, Royal Devon University Healthcare NHS Foundation Trust, Exeter, UK; 4 Department of Ophthalmology, Southend University Hospital, Southend-on-Sea, UK; 5 Faculty of Health, University of Plymouth, Plymouth, UK; 6 Department of Eye and Vision Science, University of Liverpool, Liverpool, UK

**Keywords:** Outcomes research, Health services

Correction to: *Eye* 10.1038/s41433-024-03556-6, published online 20 December 2024

In this article, incorrect graph data was provided for Figures 1 and 2. The corrected figures are as follows:
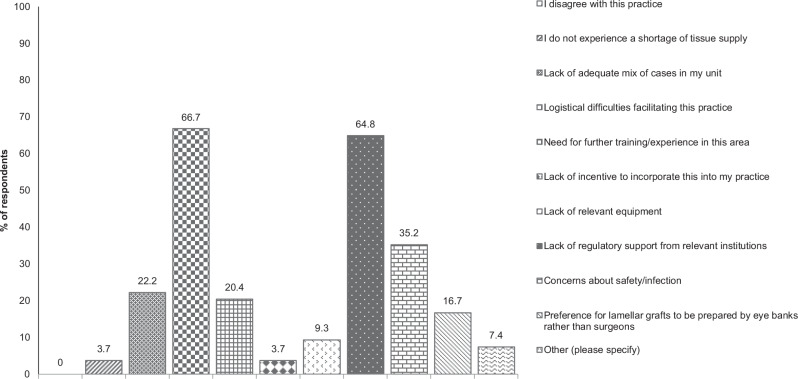
Figure 1 Bar graph of respondents’ answers to: What are the barriers to splitting donor corneas for multiple recipients in your practice? (Please tick all that apply.)


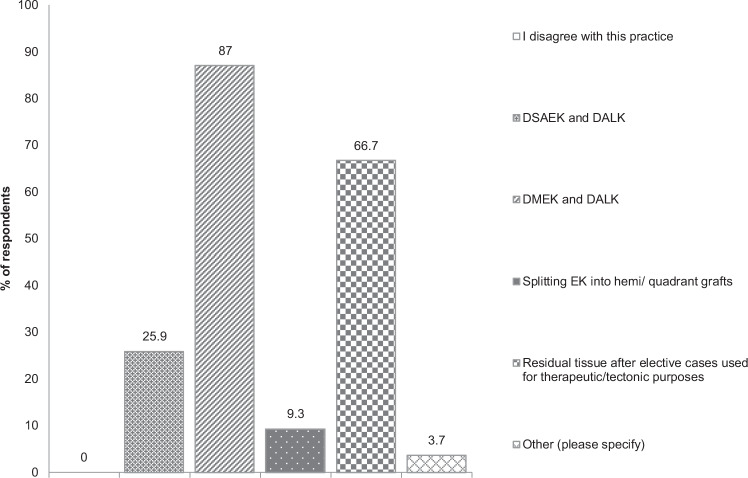


Figure 2 Bar graph of respondents’ answers to: In which ways would you prefer to split grafts? (Please tick all that apply.)

The original article has been corrected.

